# Systematic Assessment of Mortalities in Calves at Commercial Calf Ranches and the Association Between Cause of Death and Season

**DOI:** 10.3390/vetsci12101017

**Published:** 2025-10-21

**Authors:** Rebecca A. Bigelow, Phillip A. Lancaster, Brad J. White, Tera R. Barnhardt, Miles E. Theurer, Raghavendra G. Amachawadi

**Affiliations:** 1 Beef Cattle Institute, Department of Clinical Sciences, College of Veterinary Medicine, Kansas State University, Manhattan, KS 66506, USAagraghav@vet.k-state.edu (R.G.A.); 2 Heritage Vet Partners, Johnson, KS 67855, USA; 3 Veterinary Research and Consulting Services, Hays, KS 67601, USA; miles@vrcsllc.com

**Keywords:** calf ranch mortality, necropsy, respiratory disease, GI lesion, season

## Abstract

**Simple Summary:**

In the dairy industry, producers are using beef semen to produce beef–dairy calves, which has led to an increase in young calves being raised at off-site facilities known as calf ranches. However, little is known about the causes of death in these calves. In this study, researchers necropsied 243 calves that died on four ranches over a year to determine the main cause of death and whether diagnoses differed by season, sex, breed, or ranch. Each calf was carefully examined after death, and the causes were grouped as respiratory disease, digestive disease, septicemia, or other problems. Respiratory disease was the most common cause, accounting for more than two-thirds of deaths. Digestive diseases, septicemia, and other causes were much less common. Most calves only had one diagnosis, but about 10% had a secondary disease present. There were no significant differences in cause of death based on season, sex, breed, or ranch. These findings provide valuable insight into the health challenges faced by calves raised in commercial calf ranches and can help producers, veterinarians, and the industry develop better strategies to improve calf survival.

**Abstract:**

As breeding practices in dairy industry shift toward the use of beef semen, the number of calves sent off-site for rearing has increased. The limited literature describes mortalities by season, sex, or breed within calf ranches. The objectives were to identify primary and co-morbidities at necropsy in calf ranches and determine whether causes of death varied by season, sex, breed, or ranch. Systematic necropsies (n = 243) were performed monthly over 12 months at four ranches by technicians with diagnoses confirmed by veterinarians. Mortalities were classified as respiratory (RESP), gastrointestinal (GI), septicemia (SEPT), or other (OTH) based on gross necropsy findings. A subset from ranches with 12 months of necropsy data (n = 175) was analyzed using generalized linear and multinomial models to evaluate associations between RESP diagnoses or GI lesion locations and 4-month periods, sex, breed, and ranch. Respiratory disease was most common (67.5%), followed by GI (11.5%), SEPT (9.5%), and OTH (11.5%). Most (86.0%) lacked co-morbidities; RESP (7.0%) and OTH (3.7%) were the frequent primary diagnoses with co-morbidities. No significant associations were detected with time, sex, breed, or ranch (*p* ≥ 0.11 for RESP; *p* ≥ 0.13 for GI). Although inferences were limited by sample size, findings provide insight into calf mortalities and co-morbidities in commercial ranches.

## 1. Introduction

In the dairy industry, most operations traditionally raise heifers on-site after birth. However, since 2002, the percentage of operations that send their heifers off-site for rearing and development has increased threefold [1]. In 2002, 7.4% of heifers were born on-site and sent to different off-site operations. By 2014, that percentage had increased to 25.7% according to the USDA [1]. Additionally, breeding practices within the dairy industry have shifted, with more producers using semen from beef sires to produce beef–dairy cross calves raised for beef production. According to the National Association of American Breeders, the number of beef semen sales increased by 1.5 million units, while the number of dairy semen units sold decreased by 2.5 million from 2022 to 2023 [2]. Beef–dairy cross calves are economically more valuable than dairy-bred bull calves due to their improved carcass characteristics that are beneficial for beef production. However, a major issue with beef–dairy cross cattle is the increased incidence of liver abscesses (two to three times higher) compared to beef cattle [3]. Similar to dairy replacement heifers, beef–dairy cross calves are often separated from their dams as young as one day of age and shipped to an off-site operation. These facilities are often termed calf ranches or calf-rearing operations. As described in previous work, a calf ranch can be defined as an operation that rears calves from a young age, typically less than one week old, to a specific weight or age depending on the production plan for those calves [4,5].

Mortalities have been described via post-mortem necropsies in the literature for the feedlot and dairy sectors [6,7,8,9,10,11,12]. Post-mortem evaluations of dairy replacement heifers have been described previously; however, there is little published information that discusses mortalities within commercial calf ranches, specifically regarding beef–dairy cross calves [13,14,15,16]. An important aspect of health in beef–dairy cross calves is gastrointestinal lesions for a couple of reasons: (1) the influence of neonatal diarrhea on mortality and (2) the hypothesis that the primary pathogens of liver abscesses translocate the GIT through ruminal and/or intestinal lesions [17]. Magrin et al. conducted a trial in Italy investigating the association of GIT lesions found at slaughter and on-farm mortality and feeding management in “batches” of veal calves [18]. Descriptive statistics reported that more than 85% of inspected abomasa and 17% of inspected livers at slaughter showed lesions; however, the number of calves per batch was never specified. Therefore, the information is difficult to interpret externally. Thus, investigation of the gastrointestinal health in calves at commercial calf ranches is relatively lacking in the literature, especially related to identifying lesions in the gastrointestinal tract (GIT).

Bovine respiratory disease (BRD) is another significant health problem of interest in calves at commercial calf ranches. Necropsies conducted within feedlot operations report BRD as the most frequently found disease process among mortalities as well as among morbidities [19,20,21]. The temporal patterns of BRD have been explored and correlations have been made between weather/season and clinical observations of BRD [22,23,24]. For example, Cernicchiaro et al. found associations between weather variables pertaining to temperature change, mean wind chill temperature, and maximum wind speed in different lag periods, as well as interactions between these weather variables with cattle demographics, and the incidence of BRD in the first 45 days of the feeding period [23]. However, specific causal relationships between BRD and weather have not been verified. More often, authors conclude that higher incidences of BRD in the fall are more closely related to the seasonal management practices and marketing of calves typical of the North American beef production system rather than to weather [22,24,25,26]. These practices often involve weaning, commingling at marketing points where calves may be introduced to a greater number of pathogens, and movement of cattle to the feedlot, resulting in increased animal density within pens. Dairy and beef–dairy cross calves experience similar potential risk factors within the calf ranch production system, often being comingled and shipped on a truck as young as one day of age, then placed in individual hutches, introduced to a grain-based diet upon arrival, weaned from milk by 75 days of age, and eventually placed in a group pen with exposure to new animals [4,27]. Thus, investigation of potential seasonal changes in the frequency of mortalities at commercial calf ranches similar to those found in the feedlot sector is of interest.

There is published information about the incidence of BRD during different seasons within calf ranches, but the results are conflicting, where one reported higher incidence in the fall and the other in the spring [28,29]. While those studies focused on BRD morbidity, there is another study that reported about BRD cause-specific and overall mortalities in pre-weaned calves on California dairies, stating that calves treated for BRD in the spring had an increased risk of mortality compared to calves treated in the summer, and that mortalities in calves treated for BRD in the fall or winter did not differ significantly from those in the summer [30]. The season of calf mortality was not significant in their model [30]. Another study completed in California from January 1997 to October 1998 found that calves arriving in February 1998 had a twofold higher risk of dying compared to those arriving in September 1997 [31]. Identifying seasonal patterns associated with mortalities within calf ranches could allow producers to alter management practices seasonally to adjust for these effects.

Thus, the objectives of this study were to conduct systematic necropsies to document the primary and co-morbidities which occur in commercial calf ranches and to determine whether the cause of mortality changes by seasons, sex, breed, and/or calf ranches.

## 2. Materials and Methods

The Kansas State University Institutional Animal Care and Use Committee (IACUC) was contacted regarding the project, and no approval was needed because only deceased animals were evaluated.

### 2.1. Experimental Design

This cross-sectional, observational study aimed to identify the frequency of causes of mortality in commercial calf ranches, where individual animals served as the experimental unit. Four calf ranches located in the central High Plains of the United States participated in the study. Necropsies were conducted on deceased calves between 22 February 2024 and 17 January 2025, once a month at three of the four calf ranches. During the summer months, May through August, visits to each ranch were more frequent, twice a month. Necropsies at the fourth ranch took place from 22 July to 26 July 2024. Mortalities were selected for inclusion based on convenience sampling, where the technicians necropsied the calves brought to them by the operation personnel during the time they were at the ranch. Due to time constraints, not all mortalities were necropsied at each ranch. Calves were evaluated for outward signs of autolysis prior to enrollment through assessment of tissue color, smell, and texture. Cattle with severe gross autolysis did not have a necropsy performed. Descriptions of ranch management practices can be seen in Supplementary File S1.

### 2.2. Systematic Gross Necropsy Process

Comprehensive, systematic necropsies were performed by trained field technicians, and all diagnoses were reviewed and confirmed by licensed veterinarians, following methodologies previously described in feedlot cattle and calves at commercial calf ranches [5,11]. A primary diagnosis was assigned to each case, and co-morbidities were identified when present, based on the severity/chronicity of lesions. Information from each necropsy was recorded using a standardized paper form as well as a digital form that included assessments of all major organ systems and detailed descriptions of observed pathologies. For each calf, a specific individual case number was assigned, and multiple digital photographs were collected.

The necropsies were performed in a similar manner to that described in previous work [5,11]. Each evaluation began with an external inspection, noting sex, breed (dairy or dairy–beef cross), and any visible abnormalities or signs of disease, such as trauma, swelling within joints and/or limbs, or signs of musculoskeletal conditions. The calf was then fully opened for internal examination of the organs and body systems, including the oral cavity, esophagus, pharynx, larynx, trachea, lungs, heart, liver, kidneys, bladder, spleen, abomasum, rumen, and intestines (both large and small). Observed gross lesions were recorded and used to determine the final diagnosis, which was then categorized into respiratory (RESP), gastrointestinal (GI), septicemia (SEPT), or other (OTH) causes.

Gastrointestinal lesions included abnormalities of the small and/or large intestine mucosa, including ulcers, hemorrhage, thickened mucosa, necrosis, obstruction, inflammation, and/or strangulation of any portion of the intestine. Abnormalities within the abomasum such as hemorrhage or ulcers, and/or the rumen, which consisted of parakeratosis, hemorrhage, or ulcers, were also considered GI lesions. Gastrointestinal cases included findings such as inflammation, hemorrhaging of the digestive tract, bloat, peritonitis, ulcers within the GI tract, abnormal digestive contents, and/or scours. Scours were sometimes confirmed based on external evidence found on the hind quarters of the calf. Septicemia diagnoses were based on gross pathological changes in pulmonary and cardiac tissues, including the widespread presence of petechiae and/or ecchymosis.

Respiratory diagnoses were identified through gross evaluation of both the pulmonary surface and cross-sections of the lungs, with the entire lungs removed from the thoracic cavity for thorough examination. Respiratory diagnoses were further categorized into bronchopneumonia (BP), bronchopneumonia with an interstitial pattern (BIP), and aspiration or embolic pneumonia. Bronchopneumonia was characterized by findings such as pulmonary consolidation (firm lungs), fibrin adhesions, and abscesses, though not all these abnormalities were observed in every case. Chronic cases typically involved extensive lesions, widespread consolidation, abscessation, and/or adhesions. Cases that were BIP, which has been previously documented in feedyard cattle, typically presented with cranioventral bronchopneumonia and interstitial pneumonia in caudodorsal regions [11]. Aspiration or embolic pneumonia was diagnosed based on the presence of scattered pulmonary abscesses without a consistent pattern. Cases that could not be clearly categorized under GI, septicemia, or respiratory were placed in the “other” group, which included congestive heart failure, endo/myocarditis, diphtheria, hardware, umbilical infection, joint infection, or unknown causes.

### 2.3. Statistical Analysis

Descriptive statistics were performed in all cases. Statistical analyses were performed on a subset of cases. Necropsies were performed each month at three of the four calf ranches. At the fourth calf ranch, necropsies were performed only in the month of July, and thus these cases were removed from statistical analyses. To assess the effect of time on the likelihood of a RESP diagnosis or observation of GI lesions, cases for each month were categorized into 4-month periods. The first 4-month period consisted of February, March, April, and May of 2024. The second period was June, July, August, and September of 2024, and the third period included October, November, December of 2024 and January of 2025. Cases from each month were grouped in this way to ensure an equal distribution and representation of cases from each calf ranch within each time category. A generalized linear model was used to evaluate the probability of being diagnosed as RESP in calves that died using the *glm* function with the binomial family and logit link from the stats package in R studio. Period (1, 2, and 3), sex (male vs. female), breed (beef–dairy cross vs. dairy), and calf ranch (Ranch A, Ranch B, and Ranch C) were included to determine the likelihood of a RESP diagnosis for each factor. The outcome of RESP diagnosis was binomialized, where 1 corresponded to a RESP diagnosis and 0 represented any other diagnosis.

Using the *multinom* function from the nnet package in R studio, a multinomial model was used to evaluate the association of finding a GI lesion and the segment of the GI in which it was found with period, sex, breed, and calf ranch. The GI lesion outcomes were categorized as the following: 0 for no GI lesion, 1 for lesion(s) found in the upper GI tract (the rumen and/or abomasum) only, 2 for lesion(s) found in the lower GI tract (the small and/or large intestines) only, or 3 for lesions found in both the upper and lower GI tracts.

## 3. Results

Two hundred and forty-three necropsies were performed across four calf ranches between February 2024 and January 2025. Cattle demographics are shown in Table 1. The majority (67.5%; 164/243) of cases were classified as respiratory, followed by GI (11.5%; 28/243), OTH (11.5%; 28/243), and finally SEPT (9.5%; 23/243). A detailed breakdown of primary diagnoses and co-morbidities can be found in Table 2 and Table 3, respectively.

**Figure 1:**
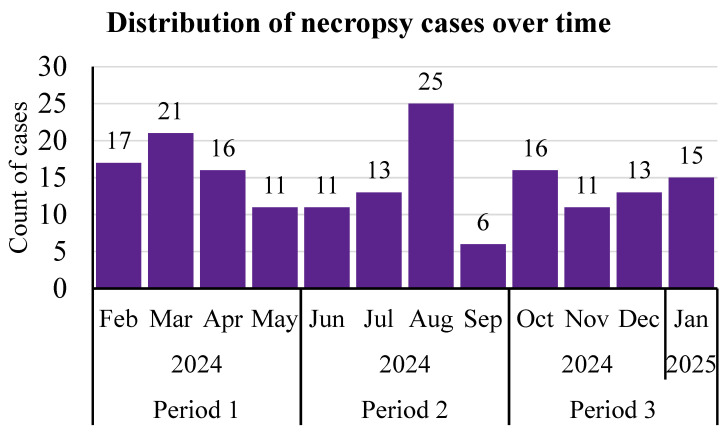
The distribution of the subset of necropsy cases (n = 175) that were included in statistical analyses by month. Cases from the fourth calf ranch were excluded from analyses due to the fact that all cases were collected in July. The association of categorizing a case as a RESP diagnosis with 4-month period, sex, breed, or calf ranch was tested, as well as the association of these factors with the probability of categorizing GI lesions by their location within the GI tract.

Descriptively, specific to the 4-month period subset (n = 175), 37.1% of cases (65/175) were necropsied in period 1, 31.4% (55/175) in period 2, and the remainder (31.4%; 55/175) in period 3 (Figure 1). Of the subset that was analyzed statistically, a majority were females (78%; 136/175). Over half of the cases (55%; 96/175) were beef–dairy cross, and the remainder were dairy calves. In period 1, 69.2% (45/64) of cases were RESP, 10.8% (7/65) were GI cases, 3.1% (2/65) were SEPT, and the remaining 16.9% (11/65) were classified as OTH. In period 2, most cases were RESP (67.3%; 37/55), followed by GI (16.4%; 9/55), then SEPT (9.1%; 7/55), and finally OTH (7.3%; 4/55). For period 3, 56.4% (31/55) of cases were RESP, 10.9% (6/55) of cases were GI, 12.7% (7/55) were SEPT, and 20.0% (11/55) were OTH.

Relative to the RESP diagnoses and the 4-month period subset, in period 1, 88.9% (40/45) of RESP cases were BP and 11.1% (5/45) were BIP. In period 2, 86.5% (32/37) were BP, 8.1% (3/37) were BIP, and 5.4% (2/37) were aspiration or embolic pneumonia. For period 3, 87.1% (27/31) were BP, 9.7% (3/31) were BIP, and 3.2% (1/31) were aspiration or embolic pneumonia. For the GI lesion categories, in period 1, 43.1% (28/65) of cases had zero GI lesions, 26.2% (17/65) had upper GI tract lesions only, 10.8% (7/65) had lower GI tract lesions only, and 20.0% (13/65) had lesions in both the upper and lower GI tract. In the second period, 45.5% (25/55) had no GI lesions, 16.4% (9/55) had upper GI tract lesions only, 14.5% (8/55) had lower GI tract lesions only, and 23.6% (13/55) had lesions in the upper and lower GI tract. In period 3, the majority (60.0%; 33/55) had no GI lesions, 20.0% (11/55) had lesions in the upper GI tract only, 12.7% (7/55) had lesions in the lower tract only, and 7.3% (4/55) had lesions in the upper and lower sections of the tract. A 2 × 2 table describing the distribution of GI lesions and their locations from all 4-month periods can be found in Table 4.

A generalized linear model was used to evaluate the probability of a RESP diagnosis in mortalities and potential associations with period, sex, breed type, and the calf ranch. A multinomial model evaluated the association of these factors with the presence and location of GI lesions in mortalities. None of the factors tested were significantly associated with the probability of RESP diagnoses (*p* ≥ 0.11) or the presence and location of GI lesion(s) (*p* ≥ 0.13). The risk of diagnosing a calf with RESP at the time of necropsy or of finding at least one GI lesion did not differ between 4-month periods, sex, breed, or calf ranches.

## 4. Discussion

In this study, most diagnoses made through gross necropsy were classified as RESP (67.5%; 164/243), followed by GI (11.5%; 28/243) and OTH (11.5%; 28/243), and finally SEPT (9.5%; 23/243). These findings are consistent with previous literature that reported RESP and GI diseases are the most common diagnoses in calf mortalities [5,13,14,16,32,33]. The most frequently reported causes of death for calves as described by the USDA National Animal Health Monitoring System (NAHMS) data were respiratory (26.9%), followed by calving-related deaths (17.8%), and digestive issues (15.4%) across both beef and dairy operations [34]. There is little published in the literature about diagnosing septicemia at necropsy, but Wilson et al. reported two cases diagnosed with septicemia out of 457 dairy calves necropsied between 2008 and 2019 at the Utah Veterinary Diagnostic Laboratory, ranging from six days to five months old [35]. However, it is important to note the differences in how diagnoses were made between the current study and Wilson et al. Wilson et al. reported that diagnoses were confirmed with numerous diagnostic tests, depending on what the attending pathologists found immediately at necropsy, whereas in the present study, diagnoses were confirmed based solely upon gross pathology present at necropsy, which is certainly a limitation. Similar to mortality, previous literature reports similar trends in calf morbidity, with the majority of illnesses being GI-related or respiratory disease [36,37,38]. Most of the cases (86.4%; 210/243) did not have a secondary co-morbidity, but the majority of co-morbidities found were categorized as OTH (7.0%; 17/243) or respiratory (4.1%; 10/243). The most common primary diagnoses that had a co-morbidity were RESP (7.0%; 17/243) and OTH (3.7%; 9/243). While co-morbidities of calf mortalities are not well-described in the literature, Pardon et al. analyzed the association between different pathological lesions like pneumonia, pleuritis, pericarditis, enteritis, abomasal ulceration, ruminal bloat, and peritonitis [39]. Concurrent enteritis and pneumonia were found in 24.2% (22/91) of necropsied calves, but the association between the diagnoses was not significant. Additionally, Pardon et al. described lesions found at necropsy in all cases (n = 91) in categories which consisted of pneumonia, enteritis, frothy ruminal bloat, peritonitis, pleuritis, pericarditis, congenital heart defects, omphalitis, and intussusception [39]; however, the categories in which lesions were recorded were not mutually exclusive, and the authors did not clearly report which lesions occurred concurrently.

In the present study, period, sex, breed, and calf ranch were not significantly associated with the probability of assigning a RESP diagnosis at the time of necropsy, nor with the chance of identifying GI lesions categorized by location within the GI tract. Dubrovsky et al. conducted a study in California to examine the associations between BRD-related mortality and dam and calf management practices [30]. That study reported the season in which the calf died was not significantly associated with mortality, which agrees with present findings. However, Dubrovsky et al. performed contrasts between seasons, comparing each season to summer as the selected reference season, and found that calves diagnosed with BRD in the spring had an increased risk of BRD-caused mortality compared to calves diagnosed in the summer, and calves diagnosed in the fall and winter did not differ significantly from those in the summer [30]. It is important to note that comparisons were not made between the other seasons (for example, spring vs. fall) and that they were truly assessing case fatality risk, as they focused on BRD-specific causes of death. Unfortunately, treatment records were not available for a large portion of the data in the present study. As stated in the introduction, seasonality has been shown to be related to BRD morbidity in feedlot cattle, but there is speculation as to whether this relationship is based on the actual seasonal patterns or the management practices that are employed during those seasons [24,25,28,29,31,33,40].

The relationship between sex and calf mortality has not been described in the literature, but there are conflicting results describing the association between BRD morbidity and sex [24]. Previous studies found that male calves were at higher risk for BRD than females [41,42,43]. In contrast, Loneragan completed a retrospective study of feedlot cattle that found higher incidence of BRD-associated mortality in beef heifers than beef steers from 1997 to 1999 [25]. Breed was also investigated in that retrospective feedlot study, and dairy animals had an increased relative risk of death from any cause compared to beef steers [25]. More relevant to the commercial calf ranch, in veal herds of young calves, mortality was higher in beef cohorts compared to dairy or crossbred cohorts [39]. In contrast, in pre-weaned calves in California dairies, breed, consisting of Holstein, Jersey, and “Other”, which consisted of other purebred breeds or crossbred calves, was not significantly associated with BRD morbidity [29]. However, it is important to note that the majority of the calves in the study conducted in California were Holsteins (71.4 ± 4.0%). While the present data has a more even distribution of breeds (55% beef–dairy cross vs. 45% dairy breeds), sample size was a limitation of this study and could affect the results of statistical analyses.

Calf ranch was also not significantly associated with RESP diagnoses or GI lesion location in the present data. It is worth noting that while the distribution of mortality diagnoses did not differ, the overall incidence of disease related to these mortality diagnoses might have differed. However, incidence was not measured as this was outside of the scope of this research. Although only three calf ranches were represented within the data, it is important to recognize that management and health practices vary greatly across operations, which is common among commercial calf ranches [4]. There could be a number of things that could affect calf morbidity and mortality regarding specific calf ranch practices, such as age of arrival, how far they were shipped, arrival protocols, and health protocols such as metaphylaxis, vaccine programs, and treatment protocols, among other things. Moore et al. investigated the influence of calf supplier on the survival of Holstein bull calves raised for beef in a calf ranch setting [31]. A calf supplier was defined as a dairy producer or calf buyer who aggregates groups of calves from multiple sources for the calf ranch. A total of 120,197 calf records from 14 different calf suppliers were analyzed, comparing all other calf suppliers to supplier 13 as the reference, because they supplied calves with the best overall survival rate. In the first three weeks following arrival, calves from all other suppliers had a higher hazard ratio of mortality compared to supplier 13, ranging from 1.7 to 6.2 [31]. Moore et al.’s study provided evidence that calf suppliers could have an effect on calf survival, so while the present data did not reflect this idea, it is important for calf ranches to consider this if they are facing a consistent pattern of mortality. It is possible that the sample size was not big enough to determine whether ranch was associated with respiratory diagnoses or GI lesion location. The number of cases recorded at each location every month varied greatly, and as a result, each calf ranch was not represented evenly across time.

The authors recognize that the lack of statistical findings could be due to the small sample size. A subset was taken from the dataset for statistical analyses due to the timeline of necropsies performed at one of the four ranches. At the fourth calf ranch, all necropsies were performed in the month of July, so these cases were removed from the analyses of period, sex, breed, and calf ranch association with RESP outcomes and GI lesion locations. It is also important to note that the number of cases necropsied varied by month, ranging from 6 to 25 cases. Additionally, there were two months when one of the three ranches did not have any cases on the day the technicians were on-site. Therefore, the months were grouped into 4-month periods to account for this in the analyses. Other limitations also include the enrollment of cases based on the availability of deceased animals during the monthly visit to each calf ranch. The technicians were not always able to necropsy all animals that died during the monthly visit to each ranch due to time constraints. Unfortunately, identification was not available for each case necropsied, resulting in lack of age or treatment information for a large portion of the cases. Another limitation to consider is the lack of laboratory confirmation of diagnoses through histopathology. This was due to budgeting, time, and labor constraints for this study. These limitations related to diagnosing cause of death based on gross findings have been discussed in previous work [5]. It can be difficult to differentiate between specific disease processes within each category (RESP, GI, etc.) without confirmatory diagnostic lab work as shown by Schmidt et al. However, necropsy diagnoses are often determined solely upon gross evaluation in the field [11]. Therefore, the data collected describing gross pathology are still valid.

## 5. Conclusions

Describing mortalities as well as co-morbidities found at necropsy within commercial calf ranches is valuable, as it provides information about disease processes that could be affecting other calves on-site. This information is essential as the number of dairies that send their calves off-site for rearing continues to increase as the calf ranch sector expands. The frequency of RESP and GI diagnoses found in this study was similar to what has been reported in previous research conducted at dairy replacement heifer-raising facilities. Seasonal differences in BRD incidence have been reported previously in feedlot cattle and calves raised in calf ranch settings, but the present study did not find an association between period and the probability of a RESP diagnosis at necropsy or with the identification of GI lesions in different locations of the GI tract. No significant associations were found between the outcomes of interest and sex, breed, or calf ranch either. The authors believe this could be due to the sample size as well as the seasonal distribution of the cases. While not statistically significant, the findings of this research provide valuable descriptive information. The information recorded on cause of death and co-morbidities contribute to the small amount of literature published surrounding mortalities at commercial calf ranches and can potentially allow producers to adjust health protocols for improved prevention and therapy of these disease processes. As the commercial calf ranch sector continues to grow, the need for further research describing and analyzing health data is essential for understanding and improving morbidity and mortality within these operations.

## Figures and Tables

**Table 1:** The proportion of mortalities by sex, breed type, and calf ranch (n = 243) with a subset of mortalities (n = 175) by 4-month period that were statistically analyzed. Necropsies were performed between 22 February 2024 and 17 January 2025.

Category		Count	Percent Total
Sex			
	Female	180	74.1%
	Male *	63	25.9%
Breed Type			
	Beef–dairy cross	152	62.6%
	Dairy	91	37.4%
Calf Ranch			
	Ranch A	74	30.5%
	Ranch B	55	22.6%
	Ranch C	46	18.9%
	Ranch D **	68	28.0%
Period			
	1	65	37.1%
	2	51	31.4%
	3	51	31.4%

* Male included steers as well as one bull calf. ** Cases from Ranch D were excluded from statistical analyses because this ranch was only visited during July, while the other ranches were visited every month for a year.

**Table 2:** The frequency of primary diagnoses by disease category for all cases necropsied (n = 243) at four commercial calf ranches. Necropsies were performed between February of 2024 and January of 2025.

Primary Diagnosis		Cases	% of Cases
Respiratory			
	Bronchopneumonia	148	60.9%
	Bronchopneumonia with interstitial pattern	13	5.3%
	Aspiration/Embolic pneumonia	3	1.2%
Gastrointestinal			
	Bloat	1	0.4%
	Scours	4	1.6%
	Peritonitis	5	2.1%
	Other GI	18	7.4%
Septicemia		23	9.5%
Other			
	Congestive heart failure	2	0.8%
	Diphtheria	4	1.6%
	Endo/myocarditis	3	1.2%
	Hardware	5	2.1%
	Joint infection	1	0.4%
	Umbilical infection	3	1.2%
	Unknown	10	4.1%
Total		243	100.0%

**Table 3:** The frequency of co-morbidities by primary diagnoses for all cases necropsied (n = 243) at four commercial calf ranches. Necropsies were performed between February 2024 and January 2025.

		Primary Diagnoses			
Co-Morbidity *		Resp	GI	Other	Septicemia
Respiratory					
	Bronchopneumonia	-	1.2% (3/243)	2.9% (7/243)	0.0%
	Embolic pneumonia	-	0.0%	0.0%	0.0%
Gastrointestinal					
	Peritonitis	0.0%	-	0.0%	0.4% (1/243)
	Other GI	0.4% (1/243)	-	0.0%	0.0%
Septicemia		0.8% (2/243)	0.8% (2/243)	0.0%	-
Other					
	Congestive heart failure	2.5% (6/243)	0.0%	0.4% (1/243)	0.0%
	Congenital defect	0.4% (1/243)	0.0%	0.0%	0.0%
	Diphtheria	0.4% (1/243)	0.0%	0.0%	0.0%
	Endo/myocarditis	1.6% (4/243	0.0%	0.4% (1/243)	0.4% (1/243)
	Hardware	0.4% (1/243)	0.0%	0.0%	0.0%
	Musculoskeletal	0.4% (1/243)	0.0%	0.0%	0.0%
None		60.5% (147/243)	9.5% (23/243)	7.8% (19/243)	8.6% (21/243)
Total		67.5% (164/243)	11.5% (28/243)	11.5% (28/243)	9.5% (23/243)

* represents categories where there were no overlap. For example, a primary RESP diagnosis with a RESP co-morbidity was not considered concurrent.

**Table 4:** A 2 × 2 table describing the distribution of GI lesions and their locations for cases (n = 175) necropsied at three calf ranches. The upper GI tract consists of the rumen and abomasum. “Yes” for the upper GI tract means a lesion was found in the rumen and/or abomasum consisting of ulcers, hemorrhage, and/or parakeratosis. The lower GI tract consists of the small and large intestines. “Yes” for lower GI tract means a lesion was found in the small and/or large intestine consisting of hemorrhage, thickened tissue, obstruction, and/or inflammation. The percentage represents the number of cases in each category out of the total number of cases.

		Upper GI Tract		
		No	Yes	Total
**Lower GI Tract**	No	86 (49%)	37 (21%)	123
	Yes	22 (13%)	30 (17%)	52
		108	67	175

## Data Availability

The datasets presented in this article were collected from cooperating entities and are not publicly available due to confidentiality and anonymity agreements.

## Supplementary Materials

The following supporting information can be downloaded at: https://www.mdpi.com/article/10.3390/vetsci12101017/s1, Supplementary File S1: Description of management practices employed at participating calf ranches related to housing, milk feeding protocol, starter feeding protocol, group pen management, and disease treatment protocols.

## Abbreviations

The following abbreviations are used in this manuscript:

USDA	United State Department of Agriculture
BRD	Bovine respiratory disease
RESP	Respiratory
GI	Gastrointestinal
SEPT	Septicemia
OTH	Other
BP	Bronchopneumonia
BIP	Bronchopneumonia with an interstitial pattern
